# Systematic Analysis of BELL Family Genes in *Zizania latifolia* and Functional Identification of *ZlqSH1a/b* in Rice Seed Shattering

**DOI:** 10.3390/ijms232415939

**Published:** 2022-12-14

**Authors:** Yan-Ning Xie, Ting Yang, Bin-Tao Zhang, Qian-Qian Qi, An-Ming Ding, Lian-Guang Shang, Yu Zhang, Qian Qian, Zhong-Feng Zhang, Ning Yan

**Affiliations:** 1 Tobacco Research Institute of Chinese Academy of Agricultural Sciences, Qingdao 266101, China; 2 Shenzhen Branch, Guangdong Laboratory of Lingnan Modern Agriculture, Genome Analysis Laboratory of the Ministry of Agriculture and Rural Affairs, Agricultural Genomics Institute at Shenzhen, Chinese Academy of Agricultural Sciences, Shenzhen 518120, China; 3 College of Agronomy, Qingdao Agricultural University, Qingdao 266109, China; 4 State Key Laboratory of Rice Biology, China National Rice Research Institute, Chinese Academy of Agricultural Sciences, Hangzhou 310006, China

**Keywords:** Chinese wild rice (*Zizania latifolia*), rice (*Oryza sativa*), seed shattering, ZlBELL transcription factor, abscission layer

## Abstract

The loss of seed shattering is an important event in crop domestication, and elucidating the genetic mechanisms underlying seed shattering can help reduce yield loss during crop production. This study is the first to systematically identify and analyse the BELL family of transcription factor-encoding genes in Chinese wild rice (*Zizania latifolia*). *ZlqSH1a* (Zla04G033720) and *ZlqSH1b* (Zla02G027130) were identified as key candidate genes involved in seed shattering in *Z. latifolia*. These genes were involved in regulating the development of the abscission layer (AL) and were located in the nucleus of the cell. Over-expression of *ZlqSH1a* and *ZlqSH1b* resulted in a complete AL between the grain and pedicel and significantly enhanced seed shattering after grain maturation in rice. Transcriptome sequencing revealed that 172 genes were differentially expressed between the wild type (WT) and the two transgenic (*ZlqSH1a* and *ZlqSH1b* over-expressing) plants. Three of the differentially expressed genes related to seed shattering were validated using qRT-PCR analysis. These results indicate that *ZlqSH1a* and *ZlqSH1b* are involved in AL development in rice grains, thereby regulating seed shattering. Our results could facilitate the genetic improvement of seed-shattering behaviour in *Z. latifolia* and other cereal crops.

## 1. Introduction

Seed shattering is an important trait that helps wild plants adapt to the natural environment and maintain population growth. In addition, seed shattering is considered one of the most important events in crop domestication [1]. Crop domestication began approximately 10,000 years ago [2,3,4] and was accompanied by a reduction in seed shattering, changes in seed shape, reduction of dormancy, increase in grain number, and improvement of plant type and fertility—all of which are important factors in artificial selection [5,6]. Research on the genetic regulatory mechanisms underlying plant seed shattering has important theoretical significance for revealing the molecular mechanisms underlying the domestication and improvement of wild plants. In addition, it has extremely high application value for the rapid domestication of wild plants and the enhancement of their yield [7,8].

In plants, the abscission zone (AZ) consists of plant organs and several adjacent cell layers separated via abscission. During plant organ shedding, the AZ differentiates into one or several layers of parenchyma cells to form an abscission layer (AL) [9]. The AL is located between the glumes and branches and consists of a group of small parenchyma cells. The cell layers adjacent to the AL have thick and lignified cell walls [10] that provide the mechanical force required for separation [11]. With the formation of the AL and protective layers, the shedding signal initiates the degradation and separation of AZ cells, leading to plant organ shedding. From an evolutionary perspective, seed shattering after plant maturation is an adaptive feature that enables wild plants to spread more seeds into the surrounding environment, which is beneficial for self-reproduction and population preservation. In agricultural production, the weakening of seed shattering facilitates crop harvest after maturity, which greatly increases crop yield and improves human welfare [12,13].

Chinese wild rice (*Zizania latifolia*, 2n = 34) is a perennial hydrophyte belonging to the Oryzeae Dum. tribe, Oryzoideae Care subfamily, and Gramineae family [14,15]. It is an important crop that is a close genetic relative of rice, second only to *Leersia* [16]. Its caryopsis is known as Chinese wild rice and has been used as a food grain for more than 3000 years in China [17]. Indeed, Chinese wild rice was one of the six grains eaten by emperors in ancient times; the other five being rice, broomcorn millet, panicled millet, wheat, and beans [18]. *Z. latifolia* grows over a wide geographical range in China, except for Tibet. It grows in lakes, ditches, ponds, rivers, and wetlands, especially in the middle and lower reaches of the Yangtze River and the Huaihe River basin [19]. Chinese wild rice is a type of whole grain that is rich in proteins, essential amino acids, fatty acids, vitamins, and various trace elements [20,21]. Li Shizhen’s Compendium of Materia Medica of the Ming Dynasty reported that Chinese wild rice was used as adjuvant therapy for diabetes and gastrointestinal diseases. Its various effects include preventing oxidative damage, improving insulin resistance, reducing lipid toxicity, controlling weight to prevent obesity, and preventing cardiovascular disease and cancer [22]. In addition, *Z. latifolia* has many excellent traits that are considered beneficial in modern rice cultivars. As a species transitioning from wild to domesticated status, it naturally retains a large number of excellent traits lost in domesticated crops [23], such as resistance to rice blasts, sturdy stalks, strong tillering, resistance to low-temperature conditions, resistance to flooding and irrigation, a fast grain filling rate, high biological yield, and higher contents of grain proteins and phenolic compounds than ordinary rice [24]. Notably, Chinese wild rice shows strong seed-shattering behaviour after maturity; as a result, the seeds rupture spontaneously and are difficult to harvest. This results in an extremely low yield, making the commercial cultivation of *Z. latifolia* extremely difficult [25]. In the early 1950s, American breeders began to artificially select Northern wild rice with low seed-shattering behaviour that was suitable for mechanised planting. Thus, they successfully achieved the commercial production of wild rice, and a large amount of wild rice is exported globally every year [26]. With the improvement of people’s quality of life, Chinese wild rice’s nutritional value and healthcare efficacy are being increasingly recognised. Therefore, *Z. latifolia* deserves further study as an economically important crop with high nutritive and medicinal value.

In recent years, several domestic and international scholars have studied plant seed shattering [10,27,28,29], providing an important reference for research on the genetic regulation of this behaviour [30,31]. Compared with wild rice, the loss of seed shattering is one of the biggest trait changes in cultivated rice and an important marker of the transition from wild to cultivated rice [8,32]. The form of the AL differs between rice cultivars: in cultivars prone to seed-shattering behaviour, the AL develops from the epidermis to near the vascular bundles; in contrast, cultivars with low seed-shattering behaviour have no AL or have an AL that is difficult to degrade. Cultivars with moderate seed-shattering behaviour either have a partially developed AL on the glumelle side of the branches or form a complete AL on the glumelle side and an irregular AL on the lemma side [11]. In these cultivars, the AL (composed of non-degradable tissue) is wider than that of cultivars whose seeds are easy to shed and narrower than that of cultivars whose seeds are difficult to shed. To date, 12 genes related to rice seed shattering have been cloned [33]. These genes encode transcription factors (TFs) or kinases that regulate seed shattering by affecting the development or degradation of the AL [32]. The *qSH1 (LOC_Os01g62920)* gene was identified by cloning the hybrid of the Indica rice cultivar ‘Kasalath’ (whose seeds shed easily) and the Japonica rice cultivar ‘Nipponbare’ (with reduced seed shedding). The *qSH1* gene explains 68.6% of the phenotypic variation in this rice population, and it is considered the main quantitative trait locus/gene regulating AL development and seed abscission in rice. *qSH1* encodes a BELL-type homeotic protein, and its coding sequence is identical between ‘Kasalath’ and ‘Nipponbare’. However, a single-nucleotide polymorphism (SNP; G/T) in the 12-kb regulatory region upstream of the initiator codon of *qSH1* changes the expression pattern of the protein-coding sequence in the AL region. Because of this change, the AL of Japonica rice plants cannot develop normally, resulting in the loss of seed shattering [27].

Gehring (1987) [34] found that mutations in some genes—such as the extremely conserved homeobox genes—can produce homologous phenotypes. Homeobox genes encode a type of transcription regulator called a homeodomain (HD), and TFs regulate the specific expression of plant genes. The TF family in plants includes NAC (NAM, ATAF, and CUC), MADS (MADS-box), ERF (ethylene response factor), and TALE (3-amino-acid-loop extension) [35,36,37,38]. The typical homeobox domain consists of 60 amino acids (except the TALE gene family, which encodes an atypical DNA-binding domain consisting of 63 amino acids) [39,40]. Based on differences in gene sequences and evolutionary history, the TALE gene family in plants can be further divided into two subfamilies: BELL (BEL1-like HD) and KNOX (KNOTTED1-like homeobox). With the rapid development of molecular biology, bioinformatics, and next-generation sequencing technology, genes in the KNOX and BELL families have been cloned and verified in different species [35]. The *KNOX* gene family is mainly involved in cell differentiation and maintenance of the shoot apical meristem [36], whereas the *BELL* gene family helps regulate plant development, hormone responses, and stress responses [41,42,43]. However, the number of members in the *BELL* gene family varies across plant species. For example, there are 4 *BELL* family genes in the bryophyte genome [38], 13 in *Arabidopsis* [44], 15 in maize [45], and 14 in rice and potato [46]. Nevertheless, the proteins encoded by these genes are highly conserved in structure, and all contain the SKY, BEL, and HD domains [47]. Various combinations of heterologous BELL–KNOX dimers with different activities are involved in regulating plant growth and development, including factors such as the maintenance of the shoot apical meristem and its boundaries, leaf development, and flowering [48,49,50]. In rice, the BELL genes *qSH1* and *SH5* participate in rice grain abscission by promoting the development of the AZ and inhibiting lignin synthesis. In addition, *SHAT1* (shattering abortion 1) and *SH4* (shattering 4) are essential regulators of AZ formation. Yoon et al. (2014) also reported that *SH5* induces the expression of *SHAT1* and *SH4*, promoting AL formation and seed shattering [51].

Yan et al. (2022) [52] performed genome collinearity analysis in *Z. latifolia* and rice to show that the *ZlqSH1a (Zla04G033720)* and *ZlqSH1b (Zla02G027130)* genes in *Z. latifolia* are orthologous to *qSH1* (*LOC_Os01g62920*) in rice. To date, no studies have analysed the BELL family TFs in the *Z. latifolia* genome or reported the function of any BELL genes. This study is the first to perform bioinformatic analysis of BELL TFs in the entire *Z. latifolia* genome using subcellular localisation and RNA-seq. We verified the functions of *ZlqSH1a* and *ZlqSH1b* by over-expressing these genes in transgenic rice. In addition, we measured the breaking tensile strength (BTS) of *ZlqSH1a* and *ZlqSH1b* over-expressing (OE) and wild type (WT) seeds after maturity, observed the structural differences in AL tissue and fracture surfaces in transgenic and WT plants, and performed RNA-seq analysis of transgenic and WT plants to determine the influence of *ZlqSH1a* or *ZlqSH1b* over-expression on gene expression in the rice grain AL. We identified and verified the functions of *ZlqSH1a* and *ZlqSH1b* in rice and discussed their effects on the development of the AL in rice grains. Our results provide theoretical support and new genetic resources for selecting *Z. latifolia* cultivars with appropriate seed-shattering behaviour that makes them suitable for commercial cultivation.

## 2. Results

### 2.1. Identification of ZlBELL Family TFs and Chromosome Localisation

**Figure 1:**
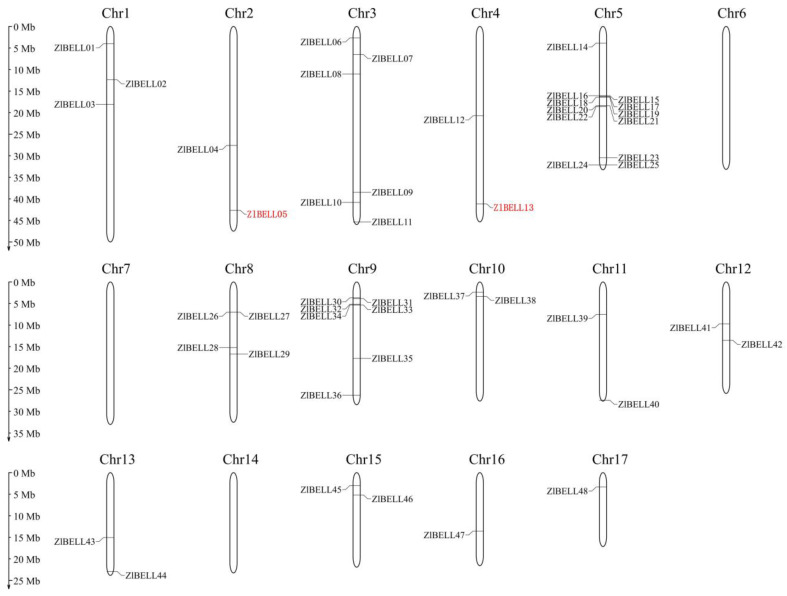
Chromosomal locations of BELL genes in *Zizania latifolia*. The gene IDs of the ZlBELL family are listed in Table S1. ZlBELL05 (ZlqSH1b) and ZlBELL13 (ZlqSH1a) are marked in red.

A total of 48 *ZlBELL* genes were identified in the *Z. latifolia* genome. Based on their location on the chromosomes, they were sequentially named *ZlBELL01–48* (Figure 1, Table S1). Notably, ZlBELL05 (Zla02G027130) and ZlBELL13 (Zla04G033720) were orthologous to qSH1 (LOC_Os01g62920) in rice and were named ZlqSH1b and ZlqSH1a, respectively [52]. The chromosome localisation analysis of *ZlBELL* genes showed that the 48 *ZlBELL* genes were distributed on 14 chromosomes in the *Z. latifolia* genome. A total of 12 genes (accounting for 25% of all *ZlBELL* genes) were located on chromosome 5. Moreover, 14.58% (7) and 12.50% (6) of the genes were located on chromosomes 9 and 3, respectively. Chromosomes 16 and 17 contained the fewest *ZlBELL* genes (only one gene each, accounting for 2.08% of the total number). The cluster of *ZlBELL* genes was located in the middle of chromosome 12 and at the top of chromosome 9 in *Z. latifolia*.

### 2.2. Phylogenetic Analysis of ZlBELL Genes

**Figure 2:**
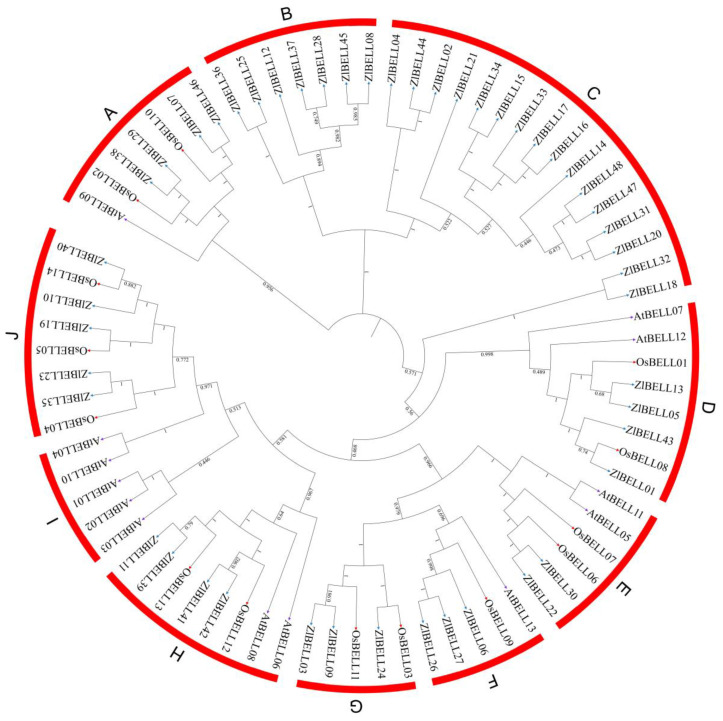
Phylogenetic analysis of BELL transcription factors in *Arabidopsis thaliana* (At), *Zizania latifolia* (Zl), and *Oryza sativa* (Os). The numbers on the major branches indicate bootstrap estimates for 1000 replicate analyses. (**A**–**J**) represent different subfamilies.

The protein sequences of 48 BELL genes in *Z. latifolia* (Table S1), 13 in *Arabidopsis*, and 14 in rice were used to analyse the BELL phylogenetic relationship between *Arabidopsis*, *Z. latifolia*, and rice. Compared with the six subfamilies of BELL genes in *Arabidopsis* (A, D, E, F, H, and I subfamilies) and seven subfamilies of BELL genes in rice (A, D, E, F, G, H, and J subfamilies), the 48 *ZlBELL* genes in *Z. latifolia* were distributed in nine subfamilies (A, B, C, D, E, F, G, H, and J subfamilies) (Figure 2). The I subfamily had the largest number (five) of *ZlBELL* genes in *Arabidopsis,* and the J subfamily had the largest number (three) in rice. The C subfamily had the largest number (16) of *ZlBELL* genes in *Z. latifolia* but did not contain *Arabidopsis* and rice *BELL* genes. The E subfamilies had fewer *ZlBELL* members (only two), and subfamily I did not contain any *ZlBELL* genes. Notably, ZlBELL05 (ZlqSH1b) and ZlBELL13 (ZlqSH1a) belonged to the D subfamily.

### 2.3. Gene Structure and Conserved Motif Analysis of Genes in the ZlBELL Family

**Figure 3:**
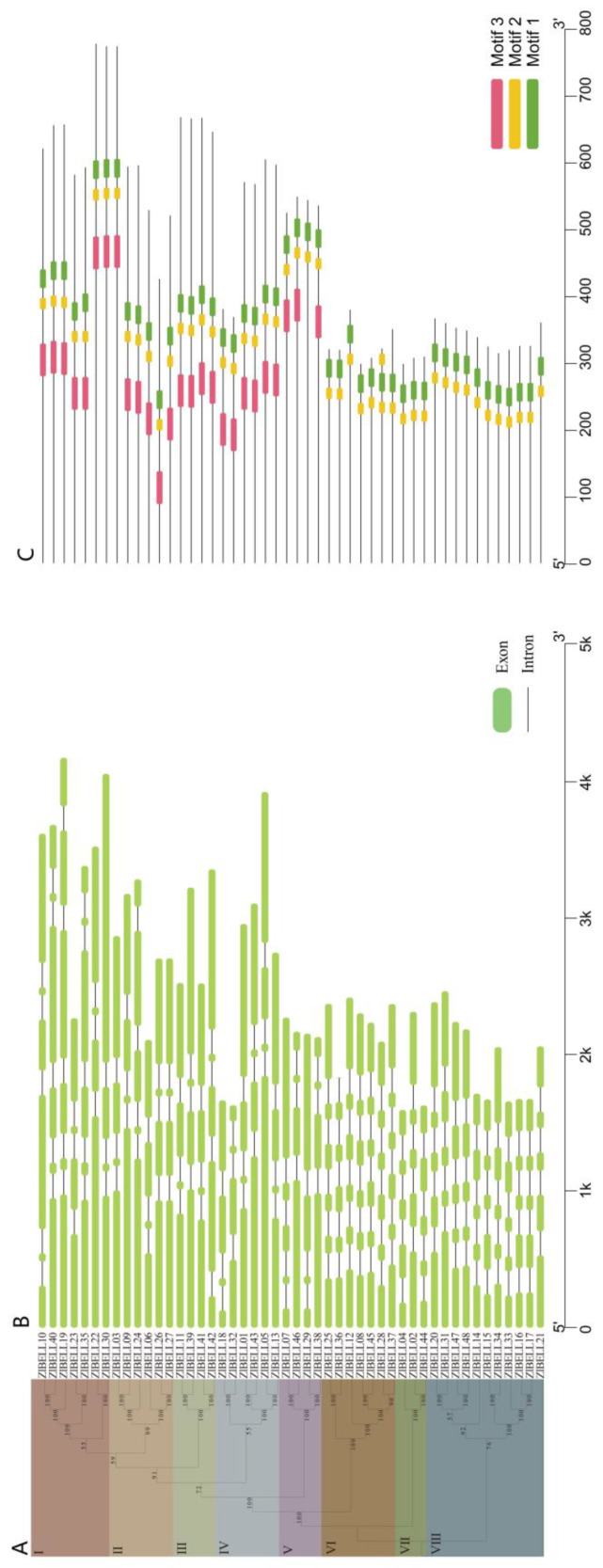
Conserved motif analysis of BELL transcription factors in *Z. latifolia*. (**A**) Phylogenetic tree; the different background colours represent different subfamilies. (**B**) Gene structure; the green boxes and black lines represent the exons and introns, respectively. (**C**) Conserved motifs; different colours represent different conserved motifs.

Phylogenetic analysis of ZlBELL protein sequences revealed that they could be divided into eight subfamilies (Figure 3A). Analysis of the intron/exon structure of each *ZlBELL* gene indicated that the exon number in *ZlBELL* genes varied from four to six; most of the genes (77.08%) contained four to five exons with a length of more than 1.6 kb. However, the intron number ranged from three to five, and did not vary greatly (Figure 3B). Protein domain analysis was performed using the MEME software to elucidate the functional diversity of *ZlBELL* proteins in *Z. latifolia*. The results showed that ZlBELL proteins had a relatively conserved structure. Three relatively conserved motifs (Motifs 1–3) were identified, of which Motifs 1 and 2 were the most conserved and were shared across all subfamilies (Figure 3C). In addition to the shared motifs, each subfamily showed some specificity; for example, Motif 3 was only present in subfamilies I, II, III, IV, and V. Notably, the typical sequence characteristics analysis of BELL family in *Z. latifolia* are shown in Figure S1. It can be seen from Figure 3 and Figure S1 that the conserved motif of the *ZlBELL* family did not completely overlap with its typical sequence.

### 2.4. Subcellular Localisation of the ZlqSH1a and ZlqSH1b Proteins

**Figure 4:**
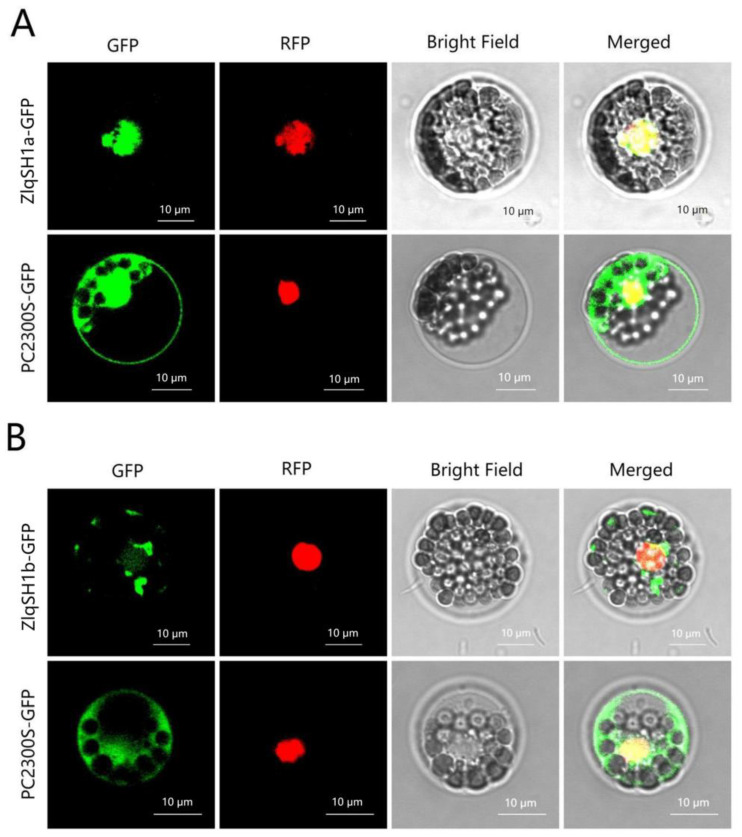
Subcellular localisation of the *ZlqSH1a* and *ZlqSH1b* proteins. (**A**) *ZlqSH1a-*GFP and PC2300S-GFP; (**B**) *ZlqSH1a-*GFP and PC2300S-GFP. GFP: green fluorescence (protein of interest); RFP: red fluorescence (nucleus); Bright: open field channel; Merged: fluorescence integration. Bar: 10 μm.

Considering the homology of ZlqSH1a (ZlBELL13), ZlqSH1b (ZlBELL05), and qSH1 (LOC_Os01g62920) [52], we chose the ZlqSH1a and ZlqSH1b for subcellular localization. To clarify the specific expression sites of the ZlqSH1a and ZlqSH1b proteins in cells, we used the PSORT program (http://psort.hgc.jp/) to predict whether they contained nuclear localisation signals. To test this prediction, we first constructed the *ZlqSH1a*-GFP and *ZlqSH1b*-GFP fusion protein over-expression constructs. The fusion expression vector, empty vector, and nuclear marker were co-transformed into rice protoplasts and cultured for 8–10 h under low light. Following this, the fluorescent signal was observed using confocal microscopy (Figure 4). The red fluorescent (RFP) regions of the nuclear marker overlapped with the green fluorescent (GFP) regions of *ZlqSH1a*-GFP and *ZlqSH1b*-GFP, indicating that both *ZlqSH1a*-GFP and *ZlqSH1b*-GFP were localised in the nucleus (Figure 4). That is, both proteins were nuclear TFs, which is consistent with the predicted results.

### 2.5. Seed Shattering Phenotype and AL-Based Histological Analysis of WT Plants and ZlqSH1a and ZlqSH1b Over-Expressing Rice

**Figure 5:**
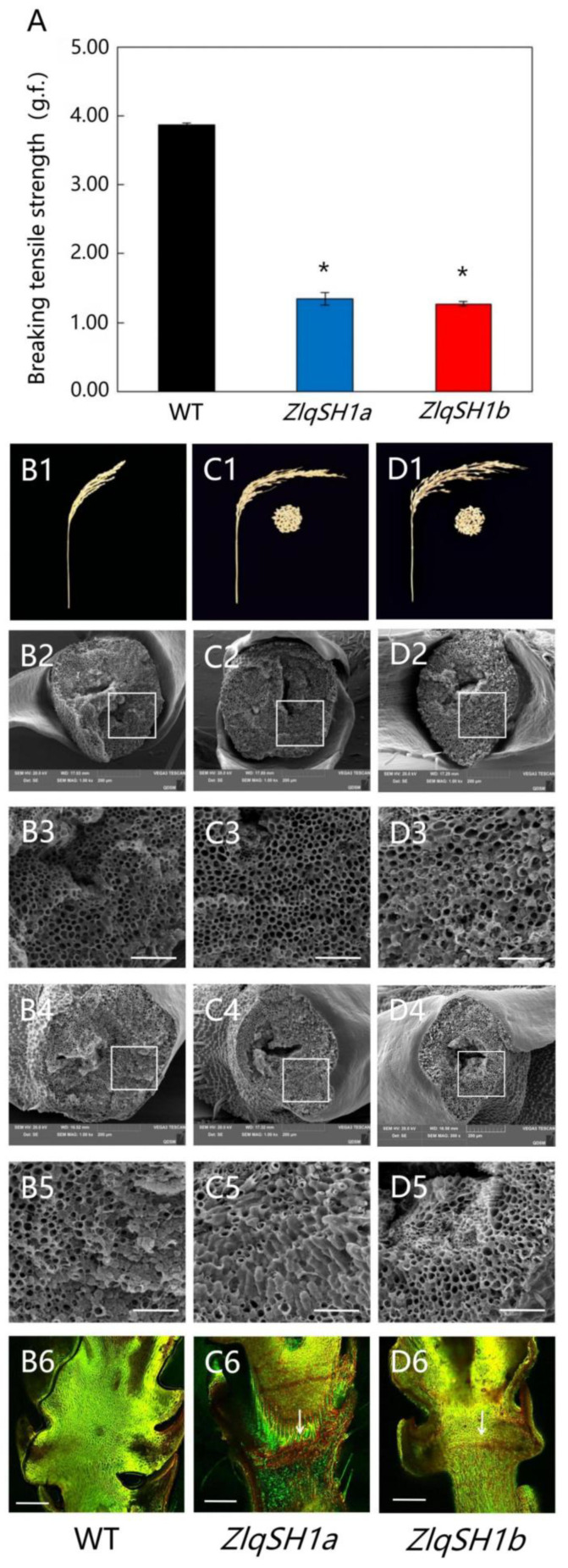
Comparison of the breaking tensile strength (BTS) and results of scanning electron microscopy (SEM) of fracture surfaces and laser confocal microscopy of the longitudinal section of the AL in wild type (WT) and *ZlqSH1a* and *ZlqSH1b* over-expressing plants. (**A**) Comparison of the BTS of wild type (WT) and *ZlqSH1a* and *ZlqSH1b* over-expressing plants 30 days after pollination. The values are means ± SD (*n* = 50 grains). The g.f. is the gravitational unit of force. Different letters denote significant differences (* *p* < 0.05) determined using Tukey’s honestly significant difference analysis. (**B1**,**C1**,**D1**) represent the panicle shape and natural seed-shattering condition of WT and *ZlqSH1a* and *ZlqSH1b* over-expressing plants, respectively. The middle part of the photo of the transgenic plants shows the seeds that were spontaneously shed that were collected in a bag. Bars = 10 cm. (**B2**,**C2**,**D2**,**B4**,**C4**,**D4**) show the scanning electron micrographs of the pedicel–seed junction after grain separation. Bars = 200 μm. (**B3**,**C3**,**D3**,**B5**,**C5**,**D5**) show close-up SEM images of regions enclosed in the white boxes. Bars = 50 μm. (**B6**,**C6**,**D6**) show fluorescence images of longitudinal sections of the pedicel–seed junction stained with acridine orange. The white arrows point to areas corresponding to the abscission layers in WT and the *ZlqSH1a* and *ZlqSH1b* over-expressing plants. Bars = 100 μm.

To verify the function of the *ZlqSH1a/b* gene, we constructed its over-expression vector and transformed it into a non-shattering rice cultivar, L422, to obtain positive transgenic rice plants. Phenotypic observations showed that at the harvest stage, *ZlqSH1a* and *ZlqSH1b* over-expressing plants had fewer seeds remaining in the rice panicle than WT plants. Thus, *ZlqSH1a/b* are speculated to be key genes for seed shattering in *Z. latifolia*. Thirty days after flowering and pollination, the BTS between the grain and the pedicel were measured in the WT and over-expressing lines using a digital tension meter. The results showed that the maximum tensile strength of the *ZlqSH1a/b* over-expressing lines was lower than that of the WT (Figure 5A). Compared with the WT, the *ZlqSH1a/b* over-expressing lines showed significantly higher natural seed shedding after maturity (Figure 5(B1,C1,D1)). Scanning electron microscopic (SEM) observations of the grain and pedicel fracture surfaces revealed that both *ZlqSH1a* and *ZlqSH1b* were involved in AL development and that the fracture surfaces were relatively smooth in the gene over-expressing plants (Figure 5(C2–C5,D2–D5)). In contrast, the fracture surfaces of the WT were rougher and more fragmented (Figure 5(B2–B5)). The results suggested that seed shattering after seed maturity was higher in *ZlqSH1a* and *ZlqSH1b* over-expressing plants, where the abscission was more complete than in the WT.

To accurately determine the histological differences in the AL between *ZlqSH1a/b* over-expressing plants and the WT, we used laser scanning confocal microscopy (LSCM) to compare the longitudinal sections of spikelets at the flowering stage. Samples from the *ZlqSH1a* and *ZlqSH1b* over-expressing plants showed a complete AL composed of small flat parenchymal cells of equal diameter between the pedicel and grain. The longitudinal section showed continuous lines, indicating cell abscission between the pedicel and the grain (Figure 5(C6,D6)). In contrast, no abscission was formed in the WT, and no single obvious line indicated cell abscission (Figure 5(B6)). These anatomical features of the AL were observed 3–5 days after the rice plant flowered, and similar features were maintained in the mature grain. Considering the natural grain shedding behaviour of *ZlqSH1a* and *ZlqSH1b* over-expressing plants, the results of BTS and SEM analysis indicated that seed shattering was significantly higher in the transgenic plants than in the WT. This was consistent with the observations of the longitudinal AL sections, indicating that *ZlqSH1a* and *ZlqSH1b* were involved in AL tissue development.

### 2.6. Effects of ZlqSH1a and ZlqSH1b Over-Expression on the Transcriptome of the AL in Rice

**Figure 6:**
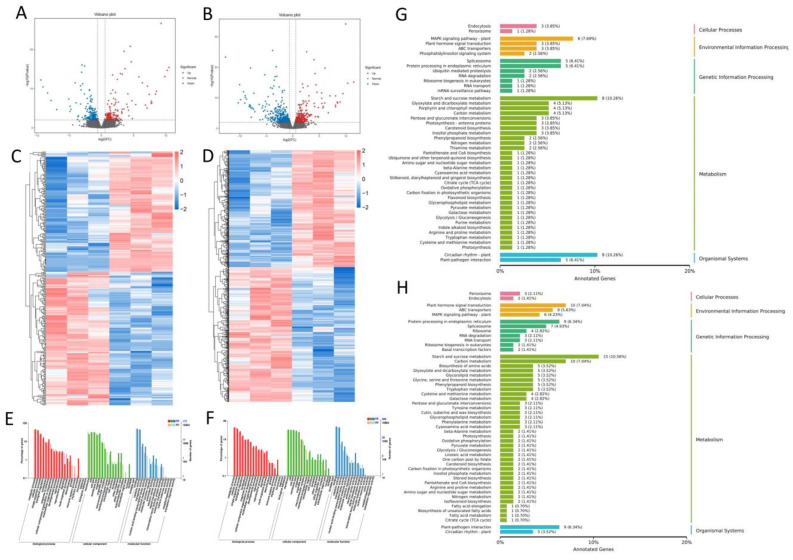
Analysis of RNA-seq results of AL tissues in wild type (WT) plants and *ZlqSH1a* and *ZlqSH1b* gene over-expressing plants. (**A**,**B**) Volcano plots of differential expression between WT and the *ZlqSH1a* and *ZlqSH1b* over-expressing plants, respectively. The green dots represent down-regulated differentially expressed genes (DEGs), the red dots represent up-regulated DEGs, and the black dots represent non-DEGs. (**C**,**D**) DEG cluster diagrams between WT tissues and tissues from *ZlqSH1a* and *ZlqSH1b* over-expressing plants, respectively. The abscissa shows the sample name and the clustering patterns of samples, and the ordinate represents the clustering patterns of DEGs and other genes. Different columns represent different samples, and different rows represent different genes. The colour represents the level of gene expression (log10-transformed) in the sample (FPKM + 0.000001). (**E**,**F**) Statistics related to the Gene Ontology (GO) annotation of DEGs in WT vs. *ZlqSH1a* and *ZlqSH1b* over-expressing plants, respectively. The abscissa shows the GO classification, the left side of the ordinate is the percentage of the number of genes, and the right side is the number of genes. (**G**,**H**) Statistics related to the Kyoto Encyclopaedia of Genes and Genomes (KEGG) annotation of DEGs in WT vs. *ZlqSH1a* and *ZlqSH1b* over-expressing plants, respectively. The ordinate shows the name of the KEGG metabolic pathway, and the abscissa shows the number (and proportion) of genes annotated under the corresponding pathway.

To analyse the gene functions of *ZlqSH1a* and *ZlqSH1b*, we performed RNA-seq experiments using AZ tissues from WT and *ZlqSH1a* and *ZlqSH1b* over-expressing plants. The unit structure of the AL consists of a 1 mm pedicel region and a 1.5 mm grain. We analysed the differential expression of genes in the two groups of samples and visualised the statistically significant differences using a volcano plot (Figure 6A,B). Hierarchical cluster analysis was performed using the selected differentially expressed genes (DEGs), such that genes with similar expression patterns were clustered (Figure 6C,D). Compared with the WT, 327 DEGs were identified in *ZlqSH1a* over-expressing plants, of which 155 were up-regulated and 172 were down-regulated (Table S2). Compared with the WT, 696 DEGs were identified in *ZlqSH1b* over-expressing plants, out of which 323 were up-regulated and 373 were down-regulated (Table S3).

The two sets of DEGs shared 172 genes. Gene Ontology (GO) and Kyoto Encyclopaedia of Genes and Genomes (KEGG) enrichment analyses were performed on these DEGs. The DEGs were searched against the GO database to classify the normalised gene function and to elucidate the cell components, biological processes, and molecular functions involved in AL development. Pathway annotation and enrichment analysis of DEGs helped further interpret the gene function. A total of 562 DEGs were assigned to three classes (Biological Process, Molecular Function, and Cellular Component) and 44 subclasses of GO (Figure 6E,F). The main subclasses were “cell part”, “cell”, and “organelle” in the Cellular Component; “metabolic process”, “cellular process”, and “single-organism process” in Biological Process; and “binding”, “catalytic activity”, and “transporter activity” in Molecular Function. All DEGs were subjected to KEGG pathway enrichment analysis to understand the complex biological processes of the transcriptome (Figure 6G,H). The results showed that 411 DEGs were annotated and enriched in several KEGG pathways, including “MAPK signalling pathway-plant”, “Starch and sucrose metabolism”, “Carbon metabolism”, and “Plant hormone signal transduction”. In this study, we mainly focused on “Carbon metabolism” and “Plant hormone signal transduction”.

### 2.7. Expression of Seed Shattering-Related Genes and Validation by qRT-PCR

**Figure 7:**
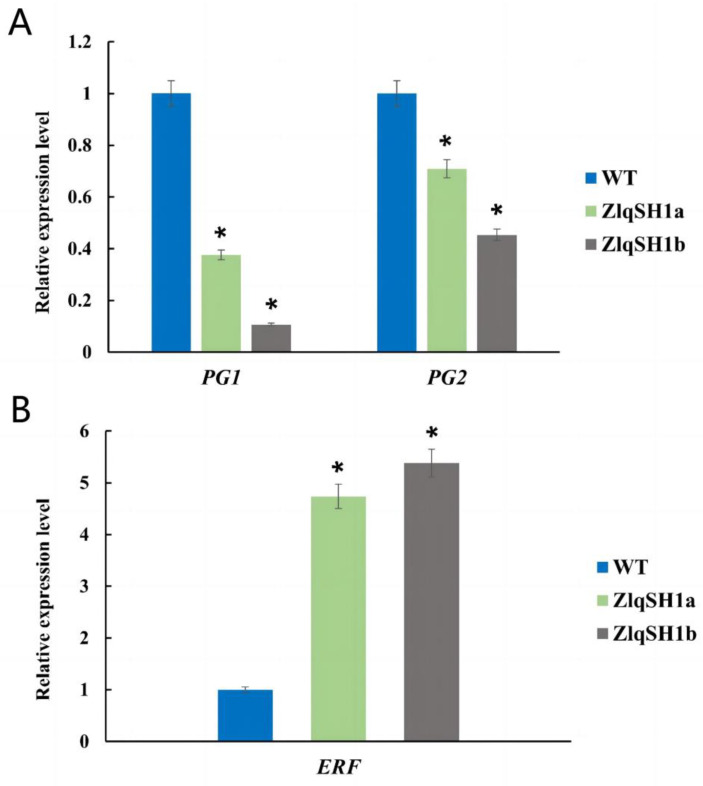
Expression of seed-shattering related genes ((**A**) PG1 and PG2; (**B**) ERF) and validation by qRT-PCR. The rice housekeeping gene UBQ5 was used as an internal control to normalize gene expression data. Two-tailed Student’s *t*-tests were used to compare the expression levels between wild-type plants and the *ZlqSH1a*/*b* over-expressing plants (* *p* < 0.05). *PG1*, polygalacturonase 1; *PG2*, polygalacturonase 2; *ERF*, ethylene-responsive transcription factor.

As an important plant hormone, ethylene can regulate the abscission of seeds and flowers [53]. Moreover, increased polygalacturonase activity is related to cell separation during fruit shedding [54]. The previous results showed that *ZlqSH1a* and *ZlqSH1b* over-expressing plants were significantly different from WT plants in terms of seed-shattering-related morphology and histology. Based on the annotations of the two sets of DEGs, we selected three seed-shattering-related genes for qRT-PCR validation. The three candidate genes—including two down-regulated genes and one up-regulated gene—were regulated in the same direction in the *ZlqSH1a* and *ZlqSH1b* over-expressing plants. The following candidate genes were included: polygalacturonase 1 (*PG1*), polygalacturonase 2 (*PG2*), and ethylene-responsive transcription factor (*ERF*). All candidate genes were expressed in the AL of WT and transgenic plants. We used the rice housekeeping gene *UBQ5* as the housekeeping gene to normalise the gene expression levels. Compared with the WT, the expression levels of *PG1* and *PG2* were significantly down-regulated in *ZlqSH1a* and *ZlqSH1b* over-expressing plants, whereas *ERF* was significantly up-regulated (*p* < 0.05) (Figure 7). This was consistent with the transcriptome sequencing results (Figure 6, Tables S2 and S3).

## 3. Discussion

### 3.1. Full-Genome Analysis of BELL Family TFs in Z. latifolia

Studies have shown that approximately 300 million years ago, at least one *BELL* gene was already present in the common ancestor of extant spermatophytes and that this gene domain is highly conserved [55]. To date, members of the BEL1-like family of TFs have been identified in every plant species studied. With the advent of full-genome sequences, the breadth and potential functions of this key family of DNA-binding proteins can now be fully understood. Genes in the *BELL* family are involved in plant vegetative growth, fruit development, and seed abscission. In apples, the BELL1-like gene *MDH1* is mainly expressed in developing and mature flowers and fruits. In *Arabidopsis* lines, the ectopic expression of *MDH1* leads to dwarfing, reduced fertility, deformation of the carpel, and deformed silique folds [56]. In rice, the *qSH1* and *SH5* genes (homologs of the Arabidopsis *PNY*) participate in seed shattering by promoting AZ development and inhibiting lignin synthesis [51].

This study is the first to systematically identify BELL TFs in *Z. latifolia*. We identified 48 *ZlBELL* genes (Figure 1, Table S1). The chromosome localisation analysis revealed that the *ZlBELL* genes were unevenly distributed on the chromosomes of *Z. latifolia*. Some *ZlBELL* genes were located at the top and bottom of the chromosome (Figure 1), which is consistent with the findings of Sharma et al. (2014) [57]. This indicates that the *ZlBELL* genes underwent a certain degree of contraction and expansion. Introns and exons can evolve through gain, loss, insertion, or deletion [58]. Here, we found that the number of exons in the *ZlBELL* gene family ranged from four to six. Most of the genes (77.08%) contained four to five exons with a length of more than 1.6 kb, whereas the number of introns ranged from three to five and did not vary greatly. Analysis of the structure of this gene family revealed the length of the introns and exons encoding key functional domains and showed the degree of conservation of their splicing mode (Figure 3). The results indicated that in *ZlBELL* genes, the insertion or loss of introns and exons was relatively stable during the evolutionary process, resulting in relatively small differences in the number of exons and introns. This ensures stability in the biological functions of these genes to a certain extent. The conserved motifs of 48 *ZlBELL* genes in *Z. latifolia* were analysed, revealing three highly conserved amino acid motifs. Most of the conserved motifs in the same subfamily were similar, indicating stability in the function of the coding protein in each subfamily. Almost all ZlBELL proteins contained Motifs 1 and 2, which were always adjacent to each other and together constituted the ZlBELL domain. The uniqueness and conserved nature of these motifs in the same subfamily also supports the evolutionary classification of the ZlBELL gene family (Figure 3C). The 48 *ZlBELL* genes in *Z. latifolia* were distributed in 9 subfamilies (Figure 3). The C subfamily contained the largest number of members (16), whereas the E subfamily contained only two members. This indicates that the members of this subfamily evolved relatively slowly, and we speculate that the function of this gene family is relatively conserved.

### 3.2. Histological Comparison of the AL between ZlqSH1a/ZlqSH1b Over-Expressing Plants and WT

Rice grains are shed at the AL, a junction between small branches. The AL consists of one or several layers of dense cells with similar morphology. Seed shattering in rice is a complex biological trait mainly controlled by the formation and fracture of the AL [28]. *qSH1* is one of the major genes controlling seed shattering in rice [27], and the locus containing this gene *(LOC_Os01g62920)* explains 68.6% of the phenotypic variation in this species. Continuous expression of *qSH1* (encoding a BELL-type homeoprotein) at the base of spikelets promotes the formation of the AL. As explained above, an SNP causes changes in the expression pattern of *qSH1* in the abscission region of ‘Nipponbare’ plants, resulting in the loss of seed shattering. Yan et al. (2022) [52] performed genome collinearity and homology analysis in *Z. latifolia* and rice and found that the *ZlqSH1a* (*Zla04G033720*) and *ZlqSH1b* (*Zla02G027130*) genes in *Z. latifolia* were homologs of the *qSH1* (*LOC_Os01g62920*) gene in rice. Based on this, we speculated that *ZlqSH1a* and *ZlqSH1b* might be related to seed shattering in *Z. latifolia*.

In the present study, we transformed *ZlqSH1a* and *ZlqSH1b* into the rice cultivar L422 (which does not exhibit seed-shattering behaviour) and constructed *ZlqSH1a* and *ZlqSH1b* gene over-expressing rice plants. We then used a digital tension meter to measure the BTS between the grain and pedicel of WT and *ZlqSH1a* and *ZlqSH1b* over-expressing plants at the same developmental period. The results showed that the maximum tensile strength was lower in *ZlqSH1a/b* over-expressing lines than in the WT (Figure 5A). In addition, the number of naturally shed seeds after maturity was significantly higher in the *ZlqSH1a/b* over-expressing lines than in the WT (Figure 5(B1,C1,D1)). SEM analysis showed that the phenotype of the AL was very different between *ZlqSH1a* and *ZlqSH1b* over-expressing lines and the WT. In particular, the fracture surfaces of the grain and pedicel were relatively smooth in *ZlqSH1a* and *ZlqSH1b* over-expressing plants (Figure 5(C2–C5,D2–D5)) and rougher and more broken in the WT (Figure 5(B2–B5)). LSCM observation of the longitudinal section of the AL revealed a complete AL composed of small and flat parenchymal cells of equal diameter between the pedicel and grain of the *ZlqSH1a* and *ZlqSH1b* gene over-expressing plants. (Figure 5(C6,D6)). In contrast, no AL had been formed in the WT plants, and no obvious line indicated cell abscission (Figure 5(B6)). These results suggest that *ZlqSH1a* and *ZlqSH1b* affect seed shattering in rice by controlling the formation of the AL. To verify the soundness of this inference, we used RNA-seq to compare and analyse the gene expression levels of ALs in *ZlqSH1a* and *ZlqSH1b* gene over-expressing plants and the WT.

### 3.3. ZlqSH1a and ZlqSH1b Are Involved in the Regulation of AL Growth and Development

We selected two groups of DEGs based on RNA-seq analysis, which revealed that 172 genes were shared among the DEGs (Tables S2 and S3). GO and KEGG enrichment analysis of these DEGs can shed light on the cellular components, biological processes, and molecular functions involved in the development of the AL. Pathway annotation and analysis of DEGs can also help interpret the functions of the genes. We selected three genes related to seed shattering for qRT-PCR validation. Phytohormones are signal molecules produced by plants that play important roles in the regulation of plant growth and development. Ethylene—a phytohormone—regulates the shedding of flowers and seeds, and elevated levels of ethylene are often associated with tissue ageing and cellular stress [53]. Ethylene is an effective inhibitor of auxin, and the auxin content of the AZ regulates the sensitivity of this site to ethylene [59]. In the present study, expression levels of the *ERF* gene were significantly higher in *ZlqSH1a* and *ZlqSH1b* over-expressing plants than in the WT (Figure 7), suggesting that *ERF* may be related to enhanced seed shattering in the transgenic plants. In addition to plant hormones, some cell wall hydrolases also influence plant organ abscission. Some studies suggest that the increased activity of polygalacturonase is related to cell separation during fruit shedding. This is because polygalacturonase promotes the hydrolysis of pectin in the cell wall, thus changing its texture and hardness [54,60,61]. The AL of several plant species show increased polygalacturonase activity before the shedding of leaves, flowers, and fruits [62], and polygalacturonase-induced cell wall degradation is an important step in the loss of cell–cell adhesion in the abscission layer [59]. In the present study, GO functional annotation revealed that the candidate genes *PG1* and *PG2* were involved in regulating polygalacturonase activity. The expression levels of *PG1* and *PG2* were significantly lower in *ZlqSH1a* and *ZlqSH1b* over-expressing plants than in the WT, indicating that these genes are regulated by *ZlqSH1a* and *ZlqSH1b* and are involved in the development of abscission. The findings suggest that *ZlqSH1a* and *ZlqSH1b* are involved in AL development in rice grains and thereby regulate seed shattering. Hence, the *ZlqSH1a* and *ZlqSH1b* genes can be exploited as key target genes in molecular breeding to cultivate new *Z. latifolia* varieties resistant to seed shattering.

## 4. Materials and Methods

### 4.1. Samples

The *Z. latifolia* plant used in this study was collected from Baimahu Village in Jinhu County, Huai’an City, Jiangsu Province (33°11′9″ N; 119°9′37″ E). *Oryza sativa* cv. L422 was provided by Hefei Jian Gu Biotechnology Co., Ltd. *Escherichia coli*-competent DH5α and T vector were purchased from Sangon Biotech (Shanghai, China) Co., Ltd. The over-expression vector 1390-UBI was provided by Hefei Jian Gu Biotechnology Co., Ltd. (Hefei, China), and the pCAMBIA1390-ubiquitin over-expression vector was provided by Wuhan Edgene Biotechnology Co., Ltd. Equal amounts of soil and water were added to each plant box, and the experiment was repeated three times.

### 4.2. Full-Genome Analysis of BELL TFs in Z. latifolia

#### 4.2.1. Identification of the BELL TFs Family in *Z. latifolia*

The protein sequence of rice BELL was downloaded from PlantTFDB (v5.0) (http://planttfdb.gao-lab.org). The full genome data of *Z. latifolia* (genomic sequence, coding sequence, and protein sequence) were downloaded from the Genome Warehouse accessed on 4 December 2021 (https://ngdc.cncb.ac.cn/gwh/Assembly/22880/show). Using the rice BELL sequence as the query sequence, the candidate *BELL* gene in the *Z. latifolia* genome was identified using BLAST. Subsequently, the hidden Markov model (HMM) of the BELL family gene was downloaded from the PFAM database (http://pfam.xfam.org). Finally, the PFAM, NCBI conserved domains (http://www.ncbi.nlm.nih.gov/Structure/cdd/wrpsb.cgi) and SMART (http://smart.embl-heidelberg.de/) databases were consulted to verify the presence of the BELL conserved domain.

#### 4.2.2. Chromosome Localisation Analysis and Phylogenetic Analysis

The location information of all *BELL* genes in the *Z. latifolia* genome was extracted, and the online tool Map Gene 2 Chromosome (v2.0) (http:/mg2c.iask.in/mg2c_v2.0) was used to draw the chromosome localisation map of the *ZlBELL* family gene. The neighbour-joining method in MEGA7 was used to construct the phylogenetic tree for the protein sequences of *Arabidopsis*, *Z. latifolia* and rice with default parameters (Test of Phylogeny: Bootstrap method; No. of Bootstrap Replications: 1000; Gaps/Missing Data Treatment: Pairwise deletion; Multiple Sequence Alignment: MAFFT v 7.5). 

#### 4.2.3. Gene Structure and Conservative Motif Analysis

The MEME (v.5.5.0) online tool (http://meme-suite.org/tools/meme; best match length: 6–50, maximum number of motifs: 10) was used to analyse the conserved motifs in the extracted BELL protein sequence. The GSDS (v2.0) system (http://gsds.gao-lab.org/index.php) was used to visualise the gene structure.

### 4.3. Gene Cloning of ZlqSH1a and ZlqSH1b

#### 4.3.1. Total RNA Extraction and Reverse Transcription

The total RNA of *Z. latifolia* leaves was extracted with the FastPure^®^ Universal Plant Total RNA Isolation Kit (RC-401, Vazyme, Nanjing, China) according to the manufacturer’s instructions. The concentration of the extracted RNA was measured using an ultramicro spectrophotometer (OSE-260, Tiangen, Beijing, China). The extracted RNA was used as a substrate for reverse transcription using the PrimeScript^TM^ Ⅱ 1st Strand cDNA Synthesis Kit. The cDNA products obtained were stored at −20 °C for later use.

#### 4.3.2. Amplification, Transformation, and Sequencing of ZlqSH1a and ZlqSH1b

The prepared cDNA was used as a template for PCR amplification with the following primers: ZlqSH1a-F and ZlqSH1a-R for ZlqSH1a; and ZlqSH1b-F and ZlqSH1b-R for ZlqSH1b (Table S4). The PCR product was subjected to agarose gel electrophoresis (AGE) to analyse the fragment sizes and to verify whether the presence of a heteroband. Subsequently, the PCR product was recovered by DNA precipitation; 10 μL of the mixture was added to 10 μL of *Taq* enzyme, heated at 72 °C for 15 min, and ligated to a ploy(A) tail. After AGE, the product was cut from the gel and recovered. The target gene was ligated to a T vector as follows: 0.5 μL of T vector, 4.5 μL of DNA, and 5 μL of the reaction buffer were added to a centrifuge tube and incubated at 4 °C overnight. The *ZlqSH1a* and *ZlqSH1b* genes were ligated to the T vector and transformed into *E. coli*-competent DH5α cells. Positive clones were screened and sequenced.

### 4.4. Subcellular Localisation of ZlqSH1a/ZlqSH1b

The *ZlqSH1a/ZlqSH1b* constructs and the empty vector PC2300S-GFP were propagated in a bacterial solution, and the plasmids were extracted. The plasmids of *ZlqSH1a/ZlqSH1b* and the empty vector PC2300S-GFP were used to co-transform the rice protoplast with the nuclear marker. After 16 h of incubation at 28 °C in the dark, GFP and RFP fluorescence was detected using an Olympus FV1000 laser scanning microscope (Olympus, Tokyo, Japan) at 488 and 543 nm.

### 4.5. Construction of ZlqSH1a/ZlqSH1b Over-Expressing Rice

#### 4.5.1. Construction of ZlqSH1a/ZlqSH1b Over-Expressing Carriers

DNA fragments containing the full-length *ZlqSH1a/ZlqSH1b* genes were amplified with primers for seamless cloning and amplification (*ZlqSH1a/ZlqSH1b-FP*, *ZlqSH1a/ZlqSH1b-RP*) (Table S4). The amplification products were detected by AGE, and the target fragments were cut under UV light and purified. The 1390-ubi vector was digested with the restriction enzyme *Bam*HI, the digested product was separated by AGE, and the linearised 1390-ubi large fragment was recovered. After recovery, the digested *ZlqSH1a/ZlqSH1b* gene fragment was ligated with the 1390-ubi vector. A 5 μL aliquot of the product was used to transform DH5α-competent cells. The next day, monoclonal colonies were selected, and the PCR primers 1390-FP and ZlqSH1a/ZlqSH1b-RP were designed for the bacterial solution (Table S4). The monoclonal colonies with positive PCR results were sent for sequencing-based verification. The single colonies with correct sequencing results were placed in LB culture medium containing kanamycin and shaken overnight at 37 °C at 200 r/min. The over-expression vector was extracted for vector transformation.

#### 4.5.2. Agrobacterium-Mediated Transformation of Rice

The activated *Agrobacterium* strain EHA105 and previously prepared rice calluses were co-cultured to facilitate infection with *Agrobacterium*. A 25 mL aliquot of the *Agrobacterium* resuspension was added to the callus and the solution was incubated for 15 min, during which the solution was shaken gently from time to time. After soaking, the resuspension was poured out, and the callus was placed in a Petri dish lined with several sterile filter papers (which absorbed the residual bacterial liquid on the surface of the callus). Next, the disposable Petri dish was lined with three sterile filter papers, and 2.5 mL of inoculating culture medium was added. The dried callus was spread evenly on the filter paper and cultured at 23 °C for 48 h in the dark. The co-cultured callus was spread evenly and sparsely in the recovery culture medium. After approximately 5 days, the callus was transferred to the screening culture medium. After 3–4 weeks, 3–5 small, fresh, resistant callus particles in good growth condition were selected from each independent transformant and transferred to the regenerative culture medium. When the seedlings grew to 2–5 cm in length, one seedling in good growth condition from each independent transformant was transferred to a root-based culture medium. The culture conditions from recovery to root-based culture were as follows: incubation at 30 ℃ under a light incubator with a 16 h light/8 h dark photoperiod.

#### 4.5.3. Identification of ZlqSH1a/ZlqSH1b Over-Expressing Rice Plants

The following primers were designed for each promoter region to identify positive transformation by the over-expression vectors: *ZlqSH1a-F* and *ZlqSH1a-R* for *ZlqSH1a* and *ZlqSH1b-F* and *ZlqSH1b-R* for *ZlqSH1b* (Table S4). The PCR reaction mixture was prepared as follows: KOD One PCR Master Mix, 25 μL; 1390-FP, 2 μL; RP-1390, 2 μL; template, 1 μL; ddH_2_O, 20 μL. The PCR program was as follows: 94 °C for 5 min; followed by 30 cycles of 94 °C for 30 s, 60 °C for 30 s, and 72 °C for 30 s; followed by 72 °C for 5 min (Figure S2).

### 4.6. Measurement of Seed Shattering

The flowering time of the rice was set after the heading had occurred. This was done to accurately quantify the changes in the seed-shattering phenotype of T2 generation of the *ZlqSH1a/ZlqSH1b* over-expressing rice plants A digital tension meter was used to measure the BTS between the grains and pedicels of the WT and over-expressing lines at the same period 30 days after flowering and pollination. A small clip was suspended on the small hook of the tension meter, and the clip was clamped to the middle part of the seed glume and pulled horizontally so that the seed and the branch separated. At this time, the tension meter showed the maximum tensile strength required in the process, and this value was recorded. The measurement was repeated 50 times, and the average value was recorded as the tensile strength of the seed.

### 4.7. Laser Scanning Confocal Microscopy

Approximately 20 spikelets were sampled from each plant at the flowering stage to observe the structure of the AZ. A longitudinal section was made manually through the junction between the flower and pedicel, and the sections were stained with acridine orange. The sections were observed using a Leica SP8 laser scanning microscope (Leica SP8; Leica Biosystems, Nussloch, Germany) at 488 and a 543 nm.

### 4.8. Scanning Electron Microscopy

Thirty days after pollination, the florets (grains) in the middle area of the inflorescence were selected. The grains (approximately 2–4 mm in length) were cut from the pedicel near the branches and stored at 4 °C in the fixative solution used for electron microscopy. The fixed samples were taken out, and the connection between the grain and the pedicel was gently broken with tweezers. The samples were glued to the metal block and observed under a JEOL JSM-840 scanning electron microscope (JEOL Ltd., Tokyo, Japan).

### 4.9. RNA-seq Analysis

The AL of WT and *ZlqSH1a/ZlqSH1b* over-expressing plants were collected 3–5 days after flowering, and RNA was extracted from the AL tissue. This step was performed for three biological replicates, with three plants in each replicate. Paired-end libraries were constructed and sequenced using Illumina HiSeq 2500. The raw reads were mapped to the reference genome (Os-Nipponbare-Reference-IRGSP-1.0, MSU7) using TopHat2 with the default parameters [63]. Cuffdiff was used to calculate the fragments per kilobase of exon of the mapped reads per million genes, and the DEGs (fold change ≥ 2) between WT and *ZlqSH1a/ZlqSH1b* over-expressing plants were identified [64]. The DEGs were functionally analysed using the agriGO and KEGG databases [65,66].

### 4.10. Data Validation by qRT-PCR

Three seed-shattering-related genes were analysed in the *ZlqSH1a/ZlqSH1b* over-expressing and WT plants using qRT-PCR analysis. cDNAs were reverse transcribed using residual RNA from the RNA-seq analysis. The synthesised cDNAs were amplified using SYBR Premix Ex Taq (TaKaRa Biotechnology Co., Ltd., Dalian, China). Quantitative RT-PCR was performed using the SYBR Green QPCR mix (Bio-Rad Laboratories, Hercules, CA, USA), and the *UBQ5* gene (NCBI accession number: LOC4341684) was used as an endogenous control to normalise gene expression. The cycling conditions included incubation for 30 s at 95 °C, followed by 40 cycles of amplification (95 °C for 5 s and 60 °C for 30 s). All samples were repeated at least three times. The primers used in the qRT-PCR are listed in Table S4.

### 4.11. Statistical Analysis

The experiments were arranged in a randomised block design. Data were collected from each experimental group containing different numbers of plants, and the mean ± SD was calculated for the total number of plants in each experiment. Data were analysed with analysis of variance (ANOVA) in IBM SPSS 18 IIBM Corp., Armonk, NY, USA) statistical software. Tukey’s multiple comparison test was used at a significance level of *p* < 0.05 to determine any statistically significant differences between treatments.

## 5. Conclusions

The present study explored the functions of two genes in the BELL family of TFs (*ZlqSH1a* and *ZlqSH1b*) in *Z. latifolia.* We found that over-expression of *ZlqSH1a* and *ZlqSH1b* was responsible for seed-shattering behaviour after the maturation of rice grains. Our findings shed light on the mechanisms underlying seed shattering in a crop plant and can facilitate the genetic improvement of *Z. latifolia* and other cereal crops.

## Data Availability

Not applicable.

## Supplementary Materials

The following supporting information can be downloaded at: https://www.mdpi.com/article/10.3390/ijms232415939/s1.
