# Supplementary material for: Systematic Analysis of BELL Family Genes in Zizania latifolia and Functional Identification of ZlqSH1a/b in Rice Seed Shattering
Source: Int J Mol Sci. 2022 Dec 14;23(24):15939. doi: 10.3390/ijms232415939 (PMC9781759; doi:10.3390/ijms232415939)
Supplement: Supplementary file 1 [file ijms-23-15939-s001.zip › Supplementary Figures.pdf]

Supplementary Figures

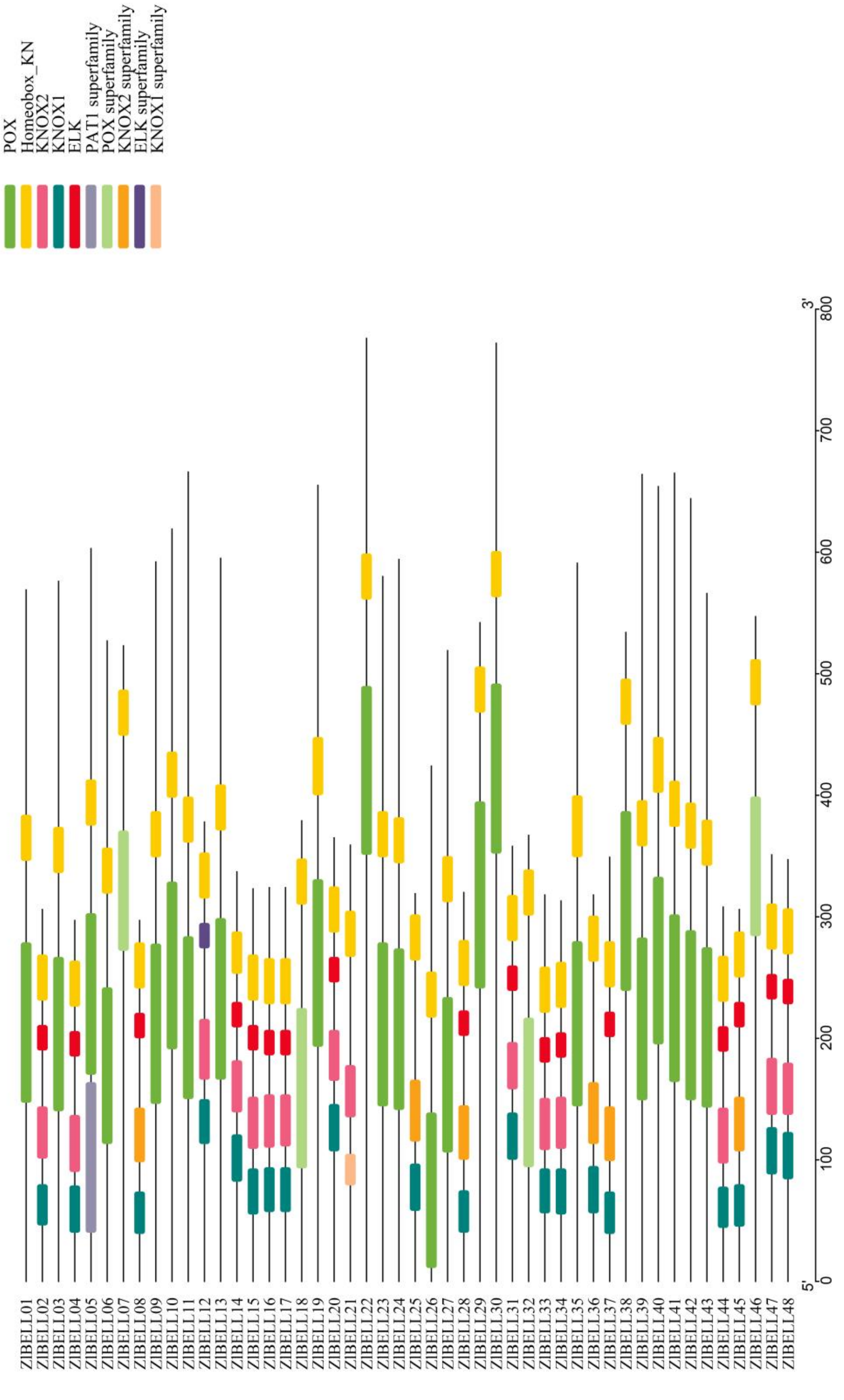

Figure S1. The typical sequence characteristics analysis of BELL family in *Z. latifolia*. Different colours represent different domains.

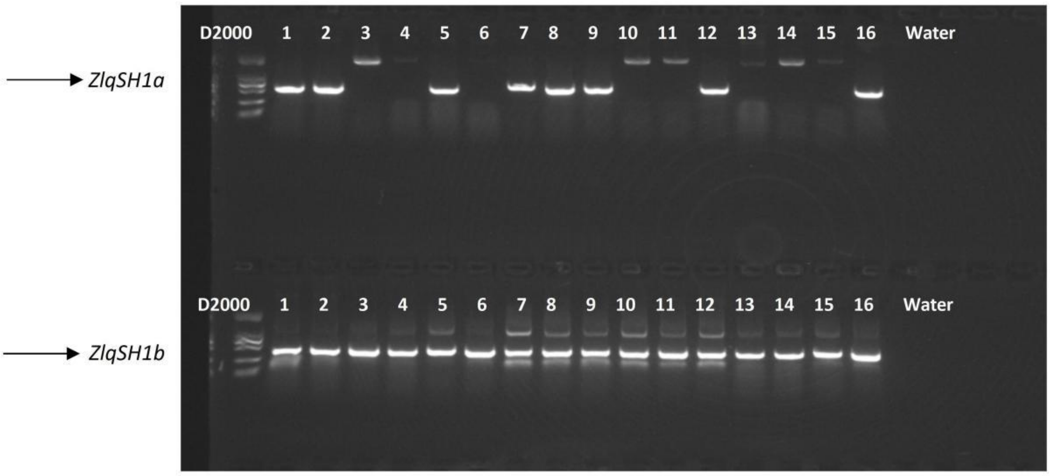

Figure S2. Screening results of transgenic positive plants.
